# Vitamin D deficiency and lower TGF-β/IL-17 ratio in a North Indian cohort of pemphigus vulgaris

**DOI:** 10.1186/1756-0500-7-536

**Published:** 2014-08-15

**Authors:** Neha Joshi, Ranjana W Minz, Shashi Anand, Nisha V Parmar, Amrinder J Kanwar

**Affiliations:** Department of Immunopathology, Post Graduate Institute of Medical Education and Research, Chandigarh, 160012 India; Department of Dermatology, Venereology, and Leprology, Post Graduate Institute of Medical Education and Research, Chandigarh, 160012 India

**Keywords:** Autoimmune bullous disease, Pemphigus vulgaris, Vitamin-D, IL-17, TGF-b

## Abstract

**Background:**

Pemphigus vulgaris (PV) is an autoimmune bullous disease caused by acantholysis of keratinocytes due to pathogenic desmoglein-3 autoantibodies. Role of vitamin D has been recently implicated in various autoimmune conditions due to its immunomodulatory effects on innate and adaptive immune responses. One of the key mechanisms of the immune regulation by vitamin D is through its anti-inflammatory effects by suppression of Th17 functions. Thus, vitamin D may be involved in pathogenesis of PV. In this study, the serum vitamin D, IL-17 and TGF-β levels in PV patients as well as healthy controls were estimated in order to understand the underlying immune mechanism involved in disease pathogenesis.

**Results:**

This retrospective study included 30 biopsy proven PV patients’ sera. Ten age matched volunteers without any cutaneous or autoimmune conditions were recruited as healthy control (HC). Serum Vitamin D levels were measured using chemiluminescence, whereas IL-17 and TGF-β levels were determined using ELISA. All patients showed deficient vitamin D levels (11.1 ± 5.8 ng/ml). Moreover, all the PV patients had elevated serum IL-17 levels (210.7 ± 105.3), whereas it was not detectable in any (n = 10) of the healthy controls sera (ELISA sensitivity ≥ 8 pg/ml). The mean serum TGF-β concentration was also lower in patient sera as compared to healthy control, and the TGF-β/IL-17 ratio was drastically reduced in patients (30.30 ± 28), as compared to healthy controls (1363.34 ± 559.52).

**Conclusions:**

Hypovitaminosis is common in North India, as ascertained by deficient levels in healthy controls, and was also consistently observed in PV patient. These low levels were not related to age or gender. The increased serum IL-17 and dramatic reduction in TGF-β/IL-17 ratio in diseased patients further indicate that dysregulation of the Treg/Th-17 axis of T effector cells may be of significance in pathogenesis of PV. Thus, the study indicates that vitamin D insufficiency may be a predisposing factor in PV, contributing through its role in any of the various adaptive immune mechanisms that regulate T cell functions *in vivo*. Thus, there is a need to further evaluate the Treg/Th-17 axis, as it may have an important role in disease progression.

## Background

Pemphigus vulgaris is an autoimmune disease which manifests with the blistering of skin and/or mucosa and may have fatal outcome. Autoantibodies against the desmosomal component desmoglein-3 are implicated in acantholysis of keratinocytes, leading to formation of clefts or bullae [1]. However, these autoantibodies are sometimes found to be absent in active disease. Moreover, they have also been at times reported in individuals without apparent disease [2]. Hence, it is postulated that various other factors such as genetic makeup and environmental triggers may contribute to disease onset. The disease pathogenesis clearly has an immunological basis. However, the exact immune mechanisms underlying the disease onset and progression are still not well understood.

T-helper (Th) cells are an essential components of host adaptive immune response, beneficial in protecting the host against infection. However, these cells can also mediate various immunological responses harmful to host such as autoimmune disorders and transplant rejection [3]. Th cells have been classified on the basis of signature cytokines secreted by them: IFNγ-secreting Th1, IL-4- and IL-5-secreting Th2 and IL-17- producing Th17 cells. Classically, the pathogenesis of autoimmune diseases were explained using Th1/Th2 paradigm, according to which some diseases manifested due to Th1 cells like Crohn’s disease, Hashimotos disease, Psoriasis, where others like SLE were thought to have a Th2 basis [4]. However, recent studies suggests that IL-17 producing Th17 cells play a crucial role in pathogenesis of various autoimmune diseases such as multiple sclerosis (MS) [5], rheumatoid arthritis (RA) [6], systemic lupus erythematosus (SLE) [7], psoriasis [8], and chronic inflammatory bowel disease [9]. Another cytokine, the role of which has gained importance in the pathogenesis of autoimmune disorders in recent times is TGF-β. It is found to be an essential differentiation factor in the homeostasis as well as in development of T regulatory (Tregs), which further inhibit inflammation and autoimmunity by counteracting the effects of other T helper cells [10]. Although there are various cytokine studies describing the Th1/Th2 cytokine levels in PV [11–13], the data on IL-17 and TGF-β is still scanty.

Vitamin D is a secosteroid, typically known for its role as a hormone involved in enhancing intestinal absorption of calcium and phosphate. In recent years, however, vitamin D has been found to have a much broader range of actions, including regulation of cell differentiation, proliferation, and apoptosis [14]. The presence of vitamin D Receptor (VDR) in human lymphocytes was one of the first observations implicating vitamin D in non calciotropic responses, and the receptor has now been described in a variety of immune cells [15]. The role of vitamin D in immune system is complex and diverse. The systemic or locally produced active form of vitamin D, 1, 25-dihydroxyvitamin D [1, 25(OH) 2D3], can exercise its effects on several immune cells, including macrophages, dendritic cells, and T and B cells. A key immuno modulatory property of 1, 25(OH) 2D3 is its ability to inhibit expression of Th1 cytokines, whilst augmenting Th2 cytokines, with 1, 25 (OH) 2D3 acting either directly via effects on T lymphocytes or indirectly via effects on antigen-presenting cells (APCs). Moreover, elevated VDR expression is also found on differentiated Th17 cells. Vitamin D provides protection against autoimmune inflammatory diseases, such as multiple sclerosis, type 1 diabetes, and IBD, partially due to its inhibitory effects on Th17 cells [16].

In recent years, a number of studies have emerged that link between vitamin D availability either from sunshine or from diet and the prevalence of autoimmune diseases. The use of supplemental vitamin D has been implicated in reducing the risk of developing multiple sclerosis [17]. Conversely, vitamin D deficiency is common in patients with autoimmune diseases. Therefore, the present study was designed to evaluate role of vitamin D deficiency and IL-17 levels in serum of PV patients in order to establish the role of these two molecules in the immune mechanisms underlying the pathogenesis of PV. TGF-β levels and TGF- β/IL-17 ratio were also analyzed to see if Treg/IL-17 balance was dysregulated in PV patients.

## Results

### Demographics

**Figure 1:**
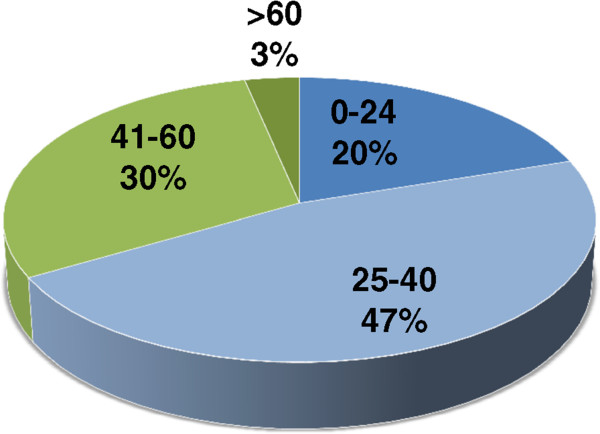
**Age distribution of pemphigus vulgaris patients**. Diagnosed patients of pemphigus vulgaris were grouped into four categories, according to their age: children and young (0–25 years), young adults (25–40 years), late adults (40–60 years) and elderly (>60 years) to establish the age at which the disease manifests.

The study included 30 biopsy proven PV patients presenting with active disease and 10 age and sex-matched controls. Age median of PV patients was 32 years (with a range of 9– 62). Table 1 summarizes the patient demographics. Figure 1 shows the age distribution of PV patients. Thus, in our study cohort, the disease is common in early adulthood, with mean age of PV patients being 34.6 ± 12.5 years and the youngest subject being 9 years old. The disease was more common in adults aged <40 years (67%) than elderly patients (33%). Also, the disease was more common in males with male to female ratio of 1.5:1.

**Table 1:** **Demographic data of pemphigus vulgaris patient and healthy controls**

	PV patients	Healthy controls	P value*
Number of subjects (N)	30	10	
Median age (years)	32	27	.1186
Age Range (years)	9-62	23-38	
Sex (F/M)	12/18	3/7	.7148

*p value > .05 considered not significant.

### Serum vitamin D levels

**Figure 2:**
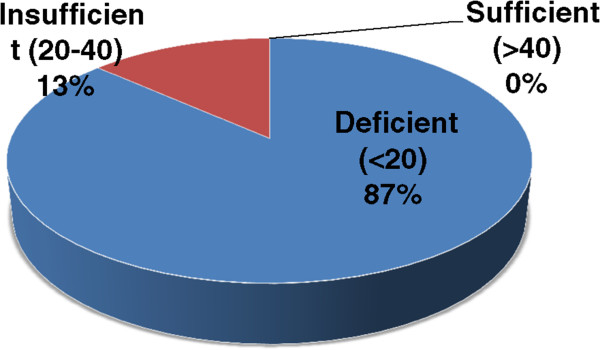
**Serum vitamin D level distribution in patients of pemphigus vulgaris.** Serum 25-hydroxy vitamin D levels were determined in patients with active disease, and levels of ≥ 40 ng/ml were considered sufficient, whereas levels of 20–40 ng/mL and < 20 ng/mL were defined as vitamin D insufficiency and deficiency, respectively.

**Figure 3:**
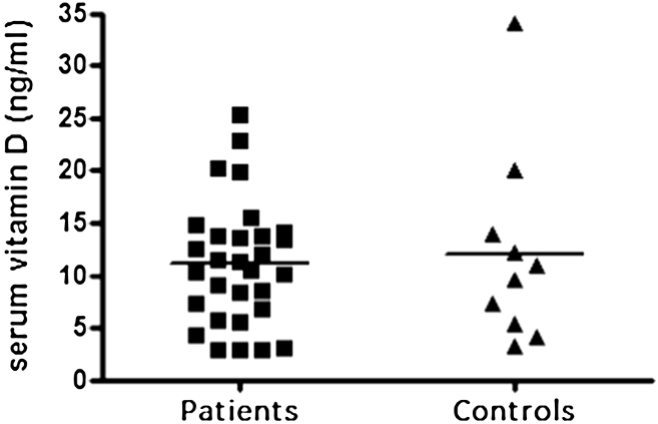
**Serum vitamin D level in patients of pemphigus vulgaris vs. control.** Serum vitamin D levels in patients and healthy controls were measured by estimatng total total serum 25-hydroxyvitamin D by electrochemiluminescence binding assay. The means were compared using unpaired *t* test and the difference was found to be not significant (p = .6958).

**Figure 4:**
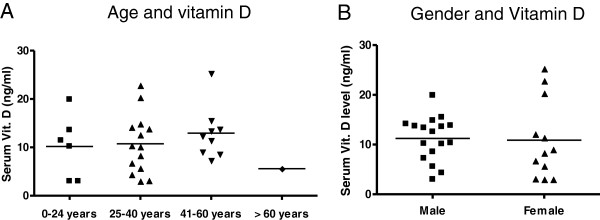
**Age and sex dependence of Serum vitamin D level in patients of pemphigus vulgaris.** To determine if the serum vitamin D levels were dependent on age, the serum levels between different age groups were compared using Kruskal-Wallis test and showed no significant difference (p = .5192) **(A)**. Similarly, no significant difference was observed in serum vitamin D levels between the two sexes (p = 0.8942) **(B)**.

We examined the total serum Vitamin D levels in patients as well as controls. The mean serum vitamin D levels in PV patients was 11.1 ± 5.8 ng/ml, with all patients showing a deficiency of serum vitamin D (range: 3–22.8 ng/ml). Figure 2 shows the serum vitamin D level distribution amongst patients of pemphigus vulgaris. As shown in Figure 3, the average serum Vitamin D levels in controls (12.1 ± 9.2 ng/ml) were not significantly different from that of patients (p = .6958). Only 1 out of the 10 healthy controls had sufficient serum vitamin D levels, suggesting that hypovitaminosis is common in the study cohort. We further analyzed if the Vitamin D levels were dependent on the age or sex of the patients and found no significant difference in the vitamin D levels between various age groups (p = .5192) or sexes (p = .7148), as shown in Figure 4.

### Serum cytokine levels

**Figure 5:**
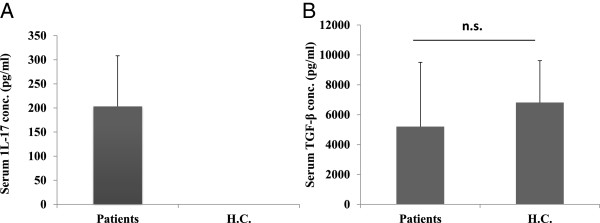
**Serum cytokine level in patients of pemphigus vulgaris vs. healthy controls.** Serum cytokine levels were measured in patients of pemphigus vulgaris and healthy control by ELISA. IL-17 was higher in patients (p < .0001) as compared to healthy control, where the levels were below the detection range (8 pg/ml). Wilcoxon Signed rank test was used, assuming the theoretical median value in healthy population to be 5 pg/ml **(A)**. TGF-β was lower in patients than healthy control, however the difference was not statistically significant (p = .2792, unpaired *t* test) **(B)**.

**Figure 6:**
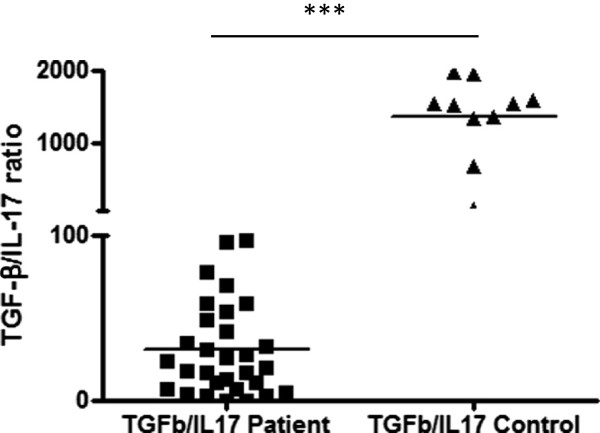
**TGF-β/IL-17 ratio in patients of pemphigus vulgaris vs. healthy controls.** Serum TGF- β and IL-17 were estimated in patients and healthy controls by ELISA and the ratios were calculated. Unpaired *t*-test was used to calculate the difference in the ratios and the ratios were found to be statistically highly significant (p < .0001).

The serum levels of IL-17 and TGF-β cytokines were examined using commercially available ELISA kits. As shown in Figure 5A, all the PV patients (n = 30) had elevated serum IL-17 levels (203.7 ± 104.7 pg/ml), whereas it was not detectable in any (n = 10) of the healthy controls sera (H.C.) (ELISA sensitivity ≥ 8 pg/ml). Moreover, the mean serum TGF-β concentration was slightly lower (5223 pg/ml) in patient sera as compared to healthy control (6817 ± 2967), although the difference was not statistically significant (p = .2792) as shown in Figure 5B. However, when we compared the ratios of TGF-β/IL-17, it was found to be drastically reduced (p < .0001) in patients (30.3 ± 28) as compared to healthy controls (1363 ± 559.52), as shown in Figure 6.

Although all patients showed vitamin D deficiency as well as elevated serum IL-17 levels, there was no significant correlation (p = .3872) between the two. Furthermore, there was no correlation between serum vitamin D and TGF-β levels, as well as IL-17 and TGF-β levels.

## Discussion

The present study demonstrates that the disease manifests at a younger age in our study group, with the youngest patient being 9 years old. Moreover, majority of patients (67%) were younger than 40 years of age. This finding is consistent with the findings complied by Kanwar *et al*. in their review stating that the epidemiology of pemphigus in India is in contrast to that from western studies that usually reported the first onset of pemphigus after 40 years of age [13]. This fact may be of significance in deciding the first line therapy, since cyclophosphamide, which has been used successfully in the management of pemphigus in India since 1986 [18], may have adverse effect on gonads, and hence, may not be suitable for adolescent and young adults. Moreover, our study indicated a male predominance. However, existing literature is abundant in studies indicating contrasting results on gender predisposition of the disease. Therefore, overall it may be reasonable to conclude that both the sexes are predisposed to this autoimmune disorder.

The present study also indicates that vitamin D deficiency is common in the North Indian population. This is in agreement with various other published reports that have evaluated serum vitamin D levels in general population of similar geographical locations of North India and Pakistan [19–21]. This indicates that despite being a sunshine abundant area, Vitamin D deficiency is common in North India. This may be attributed to a change in lifestyle that involves more indoor stay during the daytime hours. It is to be noted that all the healthy controls recruited in our study were the hospital staff, who also spent their daytime hours indoors. This explanation is further supported by the study published by Kochupillai *et al.* showing that all study groups from North India, except the one with maximum direct sunlight exposure had sub-normal concentrations of 25 (OH)vitamin D [19]. Several autoimmune diseases, including inflammatory bowel disease, MS, and type I diabetes and RA are more prevalent in population prone to Vitamin D deficiency due to inadequate sun exposure. Thus, Vitamin D deficiency may be a risk factor predisposing the North Indian population to PV, since it is established that Vitamin D can modulate the functions of a variety of immune cells, including Th-17 cells, and its role in pathogenesis of a various other autoimmune disorders is well established. Similar observations regarding vitamin D deficiency in PV patients have been reported by El-Komy *et al.*[22]*.* This is further strengthened by the observation that all our patients were deficient in Vitamin D levels during the active phase of disease as well as exhibited an increased activity of Th-17 subset, as indicated by an elevated serum of IL-17.

Until last decade, the autoimmune disorders were classified as either Th1 or Th2. However, since the discovery of IL-17, more and more studies implicating the role of Th-17 subset in autoimmune disorders have been reported. In our study, we report an increased serum IL-17(203.7 ± 104.7 pg/ml) levels as compared to controls. The levels in controls is in agreement with various other published reports that also indicate that in healthy individuals, the serum levels are usually maintained at levels <5 pg/ml [8, 23]. Thus, increased levels of IL-17 may be of significance in pathogenesis of PV, since IL-17 exhibits a variety of biological activities leading to tissue destruction during inflammation. It stimulates macrophages to produce various inflammatory cytokines, such as IL-1β and TNF-α. Expression of many genes involved in cellular adhesion, has been reported to decrease upon stimulation of keratinocytes by IL-17. Moreover IL-17 also induces the production of inflammatory mediators like nitric oxide and MMPs by keratinocytes [23]. The study also indicates a decrease in serum TGF-β levels, suggesting a compromised tolerance due to impaired function of T regulatory cells. However, the most remarkable differences were observed when we compared the TGF-β/IL-17 ratios in patients and controls, thus highlighting that the T regulatory and T effector axis has an important role in pathogenesis. These results are in agreement with findings of Xu *et al.* reporting higher Th17 cell numbers in peripheral blood as well as in lesions and lower Treg cells in pemphigus patients [24]. The hypothesis that IL-17 plays an important role in disease pathogenesis is further strengthened by the fact that the levels were also found to be high in patients on DCP therapy (data not shown) who relapsed, irrespective of their autoantibody levels as indicated by DIF. This also suggests that autoantibodies to desmosomal components may have only an initial role in inducing acantholysis, and the disease is perpetuated through other immune mediators such as cytokines. Thus, the present study firmly establishes the hypothesis that besides the classic Th1/Th2 axis, dysregulation of The Treg/Th17 axis is observed during the active form of the disease. However, to further validate this hypothesis, systematic studies in patients during various stages of disease activity are required. These studies should be designed to measure the various T cell subpopulations in lesions as well as circulation, as well as molecular studies focusing on expression of genes such as FOXP3 and RORγT during various stages of disease activity. Once well established, the gene expression ratio or cytokine levels can prove as important serological markers for monitoring disease progression, as well as to predict relapse. There is an urgent need of develop such markers, as the currently available methods such as DIF are invasive and have proved insufficient in predicting the relapse.

In the present study, since no significant difference is observed in serum vitamin D levels in patients and controls, thus, it is clear that serum vitamin D levels alone cannot account for the disease pathogenesis, and is likely to be one of the many factors underlying the complex immune mechanism operating in disease pathogenesis. This is expected since autoimmune disorders usually have a complex etiology, with various factors such as environmental as well as genetic factors contributing to disease onset. Thus, future research pertaining to role of vitamin D in PV should be designed to address the effect of vitamin D supplementation on disease outcome, effect of vitamin D treatment on various immune cells, VDR receptor expression and its polymorphism in patients of PV.

Most importantly, this study demonstrated the deranged TGF-β/IL-17 balance in PV. This paves the way for instituting anti IL-17 therapy with/without Treg modulation in future, to help the patient achieve a relapse free state.

## Conclusions

This study reaffirms that hypovitaminosis is common in North Indian population and may be a predisposing factor for autoimmune diseases onset. The study also firmly establishes that TGF-B/IL-17 axis is skewed during disease manifestation, and hence immune dysregulation plays an important role in disease pathogenesis. However, further studies including a larger patient number, and more extensive cytokine and T cell profiling are required to fully understand the exact immune mechanisms operating in disease pathogenesis.

## Methods

### Patients and controls

This retrospective study was performed using preserved sera (stored at -80°C) of 30 patient attending the dermatology Outpatient department at Post Graduate Institute of Medical Education and Research, Chandigarh, India, between May 2009 and April 2010, and diagnosed for pemphigus vulgaris (PV) by clinical, histological and immunofluorescence features. The blood samples were collected after obtaining informed consents from patients during the active form of the disease when the patients presented with skin, oral and/or mucosal lesions. Ten age-matched individuals without any autoimmune or cutaneous conditions were recruited as healthy control. The study was approved by Institution Ethics Committee of Post Graduate Institute of Medical Education and Research, Chandigarh.

### Vitamin D estimation

Serum Vitamin D levels were measured by electrochemiluminescence binding assay to measure total serum 25-hydroxyvitamin D using Roche Vitamin D total kit (Roche Diagnostics, Mannheim, Germany) on fully automated Cobas 6000 system (Roche Diagnostics, Mannheim, Germany). Serum 25-hydroxy vitamin D levels of ≥ 40 ng/ml were considered sufficient, whereas levels of 20–40 ng/mL and < 20 ng/mL were defined as vitamin D insufficiency and deficiency, respectively.

### Serum cytokine estimation

Cytokine quantification in the serum sample was done using commercially available ELISA kits for IL-17 (Peprotech, Rocky Hill, NJ USA) and TGF-β1 (BD Biosciences, San Diego, CA USA) according to manufacturer’s instructions. For the purpose of calculating the ratios, the patients with IL-17 below the detectable range were arbitrarily given a value of 5 pg/ml and those with TFG-β below the detectable range were arbitrarily given a value of 1 pg/ml

### Statistical analysis

The quantitative data is reported as mean ± standard deviation. The comparison between the cytokine levels of healthy control sera and Pemphigus vulgaris patient sera were calculated using the unpaired *t*-test. For comparing IL-17 levels of patients with healthy controls, Wilcoxon Signed rank test was used, assuming the theoretical median value in healthy population to be 5 pg/ml, since all healthy controls had value <8 pg/ml (kit sensitivity). Correlation between cytokine and vitamin D were calculated using Pearson correlation coefficient. All statistical analyses were performed using Graph Pad Prism® software.

## Abbreviations

TGF-β: Tumor growth factor-β
IL: Interleukin
TNF: Tumor necrotic factor
PV: Pemphigus vulgaris
DIF: Direct immunofluorescence
Th: T helper
Treg: T regulatory
ELISA: Enzyme linked immunosorbent assay
DCP: Dexamethasone cyclophosphamide pulse
FOXP3: Forkhead box P3
RORγT: Retinoid-related orphan receptor gamma T.
